# Associations Between Traumatic Event Exposure and Depressive Symptoms Among Chinese University Freshmen: A Conditional Process Analysis of Interpersonal Difficulties and Only-Child Status

**DOI:** 10.3390/bs16071243

**Published:** 2026-07-21

**Authors:** Fengting Wang, Xinyue Hu, Yangziye Guo, Yu Sun, Zhicheng Lu, Xue Chong, Shuang Li, Lily Wang, Guanchen Zhu, Ziwei Chen, Fuqin Mu, Yi Qiao, Min Liu, Yan Liu

**Affiliations:** 1 School of Mental Health, Jining Medical University, Jining 272013, China; 13890296451@163.com (X.H.); fuqinmu@mail.jnmc.edu.cn (F.M.); 2 School of Public Health, Jining Medical University, Jining 272013, China; gyzy2366631577@163.com (Y.G.); 13581026968@163.com (Y.S.); chongxue1053@163.com (X.C.); qiaoyi2018@mail.jnmc.edu.cn (Y.Q.); 3 School of Clinical Medicine, Jining Medical University, Jining 272067, China; lukangtong2006@163.com; 4 School of Public Health, Shandong Second Medical University, Weifang 261053, China; 19861657272@163.com; 5 Department of Community Health and Epidemiology, Faculty of Medicine, Dalhousie University, Halifax, NS B3H 4R2, Canada; lwan64@uwo.ca; 6 Faculty of Computer Science, Dalhousie University, Halifax, NS B3H 4R2, Canada; gzhu@dal.ca; 7 Faculty of Agricultural Business, Dalhousie University, Halifax, NS B3H 4R2, Canada; ziwei607863@dal.ca; 8 School of Public Health, Peking University, Beijing 100191, China; liumin@bjmu.edu.cn

**Keywords:** traumatic event exposure, depressive symptoms, interpersonal difficulties, only-child status, mediation, moderated mediation

## Abstract

Depressive symptoms are highly prevalent among university students, yet the psychosocial correlates linking exposure to potentially traumatic or severe adverse events with depressive symptoms remain incompletely understood. This study examined the association between traumatic event exposure and depressive symptoms among Chinese university freshmen, focusing on the mediating role of interpersonal difficulties and the moderating role of only-child status. A cross-sectional survey was conducted among 8079 freshmen from three universities in Shandong Province, China. Depressive symptoms were measured using the PHQ-9, traumatic event exposure was assessed using the Severe Traumatic Events Questionnaire, and interpersonal difficulties were evaluated using the Comprehensive Diagnostic Scale of Interpersonal Relationships. Mediation and moderated mediation analyses were performed using Hayes’ PROCESS macro (Model 15), controlling for sex, age, and residence. Results showed that traumatic event exposure was positively associated with depressive symptoms (β = 0.194, 95% CI [0.169–0.221]). A statistically significant indirect association through interpersonal difficulties was observed (indirect effect = 0.110, 95% bootstrap CI [0.097–0.124]). Only-child status moderated both the direct association between traumatic event exposure and depressive symptoms and the association between interpersonal difficulties and depressive symptoms. These findings suggest that interpersonal difficulties may represent an important factor associated with the relationship between traumatic event exposure and depressive symptoms, and that only-child status may influence the strength of these associations among university students.

## 1. Introduction

Depression is a serious mental health issue affecting hundreds of millions of people worldwide. Among various populations, university students are particularly vulnerable to depression due to the complex developmental challenges encountered during this transitional period of their lives, including academic pressures, social adaptation, and interpersonal conflicts (Huang et al., 2016; Yuan et al., 2022). The prevalence of depressive symptoms among college students ranges from 15% to 30%, which is substantially higher than that observed in the general population (Gao et al., 2020; Ibrahim et al., 2013). Chinese university students, in particular, exhibit increasing rates of depression, with recent studies reporting prevalence rates as high as 28.4% (Gao et al., 2020). Depressive symptoms not only impair academic performance and social functioning but also increase risks of suicidal ideation and chronic mental health disorders (Conejero et al., 2018; Mojs et al., 2015). Understanding the correlates underlying depressive symptoms in this population is thus imperative for guiding future longitudinal research on potential intervention strategies.

Traumatic events are defined as experiences that significantly affect an individual’s psychological and physiological well-being, including natural disasters, physical and emotional abuse, sexual trauma, etc. (Vrana & Lauterbach, 1994; Vrijsen et al., 2017; Wang et al., 2020). Studies have found that violence, injury, and other directly experienced traumatic events can increase the severity of depression (Infurna et al., 2016; Liu et al., 2025; Sezgin & Punamäki, 2021). The number of traumatic events is also related to the severity of depression; individuals who experience multiple traumatic events are more likely to develop severe depressive symptoms (Tracy et al., 2014). The relationship between experiencing traumatic events and the onset of depression can be explained by the diathesis-stress model. This model posits that the interaction between an individual’s genetic predisposition and environmental stressors contributes to the development of mental illness (Nielsen et al., 2020). In this model, traumatic events are considered as intense sources of environmental stress, which can activate an individual’s latent vulnerabilities, thereby increasing the risk of developing depression (Crouse et al., 2024). Following a traumatic event, individuals may experience social isolation or the breakdown of interpersonal relationships, factors that can further exacerbate depressive symptoms (Hernandez et al., 2016).

Interpersonal difficulties may be an important psychosocial pathway linking traumatic event exposure to depressive symptoms. Traumatic experiences may impair interpersonal functioning by increasing mistrust, social withdrawal, interpersonal sensitivity, negative self-perceptions, and difficulties in emotion regulation (Dong & Cao, 2025; Hughesdon et al., 2021; Kuhl & Boyraz, 2016). Students who have experienced traumatic events may therefore find it more difficult to establish supportive and stable interpersonal relationships during the transition to university (Lawrence, 2020). Poorer interpersonal relationships may in turn reduce perceived support, increase loneliness and interpersonal stress, and contribute to depressive symptoms (N. Li et al., 2025). Previous studies have shown that depressive symptoms are closely related to feelings of isolation and burden in interpersonal relationships (Prizeman et al., 2023). Among adolescents, interpersonal sensitivity and social stress are also considered important factors in the development of depression (Starr et al., 2014). However, whether interpersonal difficulties mediate the link between traumatic event exposure and depression among Chinese college students remains understudied.

Only-child status may further affect the associations among traumatic event exposure, interpersonal difficulties, and depressive symptoms. In China, the former one-child policy created a large population of young adults who grew up without siblings (H. Li et al., 2018). More broadly, declining fertility rates and smaller family sizes have become increasingly common in many societies, making sibling status and family structure relevant to youth mental health beyond the Chinese context (Downey & Cao, 2023; Vollset et al., 2020). Students from single-child families may receive more parental attention and family resources, which may support self-esteem and academic development (Chu et al., 2015). However, they may also experience greater parental expectations and have fewer sibling-based opportunities for everyday conflict resolution, emotional sharing, and peer-like family interaction (Cameron et al., 2013; Pike et al., 2005; Wikle et al., 2019; Yang et al., 2017). These developmental differences may influence how students respond to traumatic experiences and interpersonal stress. Therefore, only-child status was examined as a moderator in the present study.

**Figure 1:**
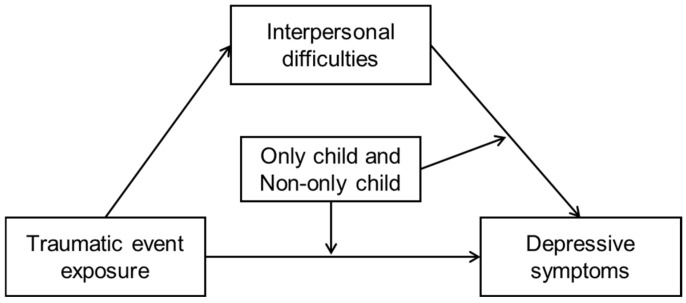
The hypothetical moderated mediation model.

Taken together, previous research has shown that traumatic or highly stressful experiences are associated with depressive symptoms, and that interpersonal relationships play an important role in students’ psychological adjustment (Wei et al., 2021). However, less is known about how traumatic events are associated with depressive symptoms among Chinese university freshmen through interpersonal difficulties, and whether these associations differ according to only-child status. Based on previous findings and the theoretical framework described above, this study constructed a moderated mediation model (Figure 1) to examine the association between traumatic event exposure, interpersonal difficulties, and depressive symptoms among Chinese first-year university students. Given the cross-sectional nature of the data, the model was used to examine associations consistent with the hypothesized framework rather than to establish temporal or causal pathways. We proposed the following hypotheses:

**Hypothesis** **1.** *Traumatic event exposure is positively associated with depressive symptoms among Chinese university freshmen.*

**Hypothesis** **2.** *Interpersonal difficulties mediate the association between traumatic event exposure and depressive symptoms.*

**Hypothesis** **3.** *Only-child status moderates the association between traumatic event exposure and depressive symptoms.*

**Hypothesis** **4.** *Only-child status moderates the association between interpersonal difficulties and depressive symptoms.*

## 2. Methods

### 2.1. Study Population

This cross-sectional study was conducted between April and October 2018 among first-year university students in Shandong Province, China. Participants were recruited from three campuses located in Jining, Rizhao, and Weifang using a cluster sampling method. The clusters were defined as intact teaching units within the participating campuses, such as selected first-year classes or majors. After the clusters were selected from the three participating campuses, all eligible first-year students within these clusters were invited to participate in the survey. The students represented a range of academic disciplines and came from diverse family residential backgrounds, including urban areas, rural counties, and villages. A total of 9928 first-year students were invited to participate. After excluding incomplete or invalid responses, 8079 valid questionnaires were retained, yielding an effective response rate of 81.38%. Among the included participants, 3225 were male and 4854 were female. The mean age of the participants was 18.36 years (SD = 0.86), with a range from 15 to 23 years. In terms of family structure, 3087 students were from single-child families, whereas 4992 students had siblings.

### 2.2. Data Collection

Data collection was conducted in the libraries of the three participating campuses. The survey was administered using a computer-based self-administration system installed on designated computers at the study sites. The system provided standardized instructions and included logical checks and skip patterns to improve response completeness and data quality. Participants completed the questionnaires independently and anonymously within a specified time period. Six trained investigators were present during data collection to provide procedural guidance and answer questions without influencing participants’ responses. To protect anonymity, no personally identifiable information was included in the analytic dataset. Each participant was assigned a unique study identification code to facilitate data management and prevent duplicate submissions. After completion, responses were uploaded directly to a secure local server at Jining Medical University. Participation was voluntary, and all participants provided informed consent before completing the survey. The study protocol was approved by the Medical Research Ethics Committee of Jining Medical University, Jining, China (approval number: 2019-JS-004), and was conducted in accordance with the Declaration of Helsinki.

### 2.3. Measurements

#### 2.3.1. Depressive Symptoms

In this study, depressive symptoms were assessed using the Patient Health Questionnaire-9 (PHQ-9), a widely used self-report instrument originally developed by Kroenke, Spitzer, and Williams to assess depressive symptom severity (Kroenke et al., 2001). The PHQ-9 consists of nine items, and each item is scored on a 4-point Likert scale, with response options ranging from 0 (not at all) to 3 (nearly every day). The total score ranges from 0 to 27, with higher scores indicating greater severity of depressive symptoms. Based on the total score, depression severity is classified as minimal (0–4), mild (5–9), moderate and severe (10 or above). Chinese version of the PHQ-9 has been widely used in Chinese populations and has demonstrated good reliability and validity (Zhang et al., 2013). In this study, the Cronbach’s α for the PHQ-9 was 0.83.

#### 2.3.2. Traumatic Event Exposure

Traumatic event exposure was assessed using the Severe Traumatic Events Questionnaire (STEQ), which was developed for the present survey to assess exposure to traumatic experiences among Chinese university students. The STEQ was constructed with reference to three established trauma exposure instruments: the Trauma History Questionnaire (THQ) (Hooper et al., 2011), the Traumatic Events Questionnaire (TEQ) (Vrana & Lauterbach, 1994), and the Traumatic Life Events Questionnaire (TLEQ) (Kubany et al., 2000). Events were considered for inclusion in the STEQ if they represented severe adverse experiences that could involve injury, abuse, bereavement, accidents, disasters, or substantial psychological distress in the Chinese university-student context.

Item development followed a structured multi-stage process. First, an initial item pool was generated based on the above validated instruments and adapted to reflect culturally relevant experiences in the Chinese university context. Second, the preliminary item pool was reviewed by a multidisciplinary expert panel consisting of specialists in psychiatry, clinical psychology, public health, and epidemiology. Experts evaluated each item in terms of clarity, cultural relevance, and content relevance. Based on their feedback, items were revised to improve comprehensibility and appropriateness for the target population. Third, a pilot test was conducted among a small sample of university students to ensure that all items were easily understood and that no ambiguous wording remained. Minor modifications were made based on pilot feedback prior to final data collection.

The final STEQ consists of 23 dichotomous items (0 = no, 1 = yes), assessing whether participants had ever experienced each event prior to the survey. Participants were considered to have experienced a specific event if they endorsed the corresponding item. The total STEQ score was calculated by summing all endorsed items, with higher scores indicating greater cumulative exposure to event types. The total cumulative exposure score was used because the present study aimed to examine the overall burden of severe adverse experiences rather than the effects of specific event categories. However, the factor structure and convergent validity of the STEQ were not evaluated in the present dataset. The assessed event categories included bereavement, serious illness, disasters, physical attack or injury, witnessing serious adverse events, childhood physical abuse, emotional maltreatment, neglect, gender discrimination, campus bullying, sexual harassment or assault, adoption/foster care, and left-behind child experiences. The full list of STEQ items is provided in Appendix A. In the present study, the STEQ demonstrated acceptable internal consistency, with a Cronbach’s alpha of 0.81.

#### 2.3.3. Interpersonal Difficulties

Interpersonal difficulties were assessed using the Comprehensive Diagnostic Scale of Interpersonal Relationships (CDSIR), developed by Professor Zheng Richang (Zheng, 1999). The CDSIR is a self-report measure consisting of 28 items designed to assess the level of interpersonal difficulties experienced by individuals. Participants responded to each item with a score of 0 or 1, representing “no” or “yes,” respectively. The total score ranges from 0 to 28, with higher scores indicating greater interpersonal difficulties. In this study, the Cronbach’s alpha was 0.89.

### 2.4. Demographic Characteristics

A self-developed general information questionnaire was used to collect demographic characteristics of freshmen, such as gender, major, place of residence (rural or urban) and family structure (only child or non-only child).

### 2.5. Statistical Analysis

Statistical analyses were performed using SPSS 24.0. To assess for potential common method bias, Harman’s single-factor test was conducted (Podsakoff et al., 2003). Descriptive statistics and independent samples t-tests were employed to analyze sociodemographic characteristics. Pearson correlation analysis was used to examine the relationships among the key study variables. The SPSS PROCESS macro Model 4 and PROCESS Model 15 were applied to test the mediation and moderated mediation models (Hayes, 2013). Only-child status was coded as 0 = non-only-child and 1 = only-child. Dichotomous variables were entered as indicator variables and were not mean-centered. Continuous variables, including traumatic event exposure and interpersonal difficulties, were standardized before the interaction terms were created. Missing data were handled by listwise deletion in the regression, mediation, and moderated mediation analyses.

Sex, age, and residence were included as covariates because these demographic characteristics are commonly associated with trauma exposure, interpersonal functioning, and depressive symptoms among university students, and including them helped reduce potential demographic confounding. Age was entered as a continuous covariate. The significance of indirect effects was tested using 5000 bootstrap samples, and 95% bootstrap confidence intervals were reported. Conditional indirect effects were estimated separately for only children and non-only children, and the index of moderated mediation was reported to evaluate whether the indirect association differed by only-child status.

## 3. Results

### 3.1. Common Method Bias Test

To assess the potential for common method bias, Harman’s single-factor test was employed. The results revealed that 15 factors with eigenvalues greater than 1 were extracted, and the first factor accounted for 15.617% of the total variance. This value is well below the critical threshold of 40%, indicating that common method bias is not a significant concern in this study. To corroborate the robustness of the regression analysis, variance inflation factor (VIF) values were examined. All VIF values were found to be below 5, confirming the absence of multicollinearity among the variables. These findings collectively demonstrate that the sample data are valid and suitable for subsequent statistical analysis.

### 3.2. Preliminary Analysis

Table 1 presents comparisons of scores for traumatic event exposure, interpersonal difficulties, and depressive symptoms across different demographic groups. Males reported slightly higher traumatic event exposure than females (*t* = 2.017, *p* = 0.044), whereas females reported higher depressive symptoms (*t* = −2.947, *p* = 0.003) and interpersonal difficulties (*t* = −3.162, *p* = 0.002). Compared with urban students, rural students reported slightly higher traumatic event exposure (*t* = −2.078, *p* = 0.038) and interpersonal difficulties (*t* = −3.875, *p* < 0.001), whereas depressive symptoms did not differ significantly by residence (*t* = −0.939, *p* = 0.348). Non-only children reported slightly higher traumatic event exposure (*t* = −2.013, *p* = 0.044) and interpersonal difficulties (*t* = −3.227, *p* = 0.001) than only children, but no significant difference was observed in depressive symptoms by only-child status (*t* = −0.785, *p* = 0.432). Overall, the magnitude of these demographic group differences was very small, with all Cohen’s d values smaller than 0.10 in absolute value. To provide additional context for the trauma exposure variable, the frequencies and percentages of each STEQ item are presented in Supplementary Table S1.

### 3.3. Correlation Analysis

The means, standard deviations and bivariate correlation coefficients for the study variables are presented in Table 2. Traumatic event exposure was positively correlated with interpersonal difficulties (r = 0.237, *p* < 0.01) and depressive symptoms (r = 0.193, *p* < 0.01). Interpersonal difficulties were also positively correlated with depressive symptoms (r = 0.481, *p* < 0.01), indicating significant associations among the study variables.

### 3.4. Mediation Effects Analysis

**Figure 2:**
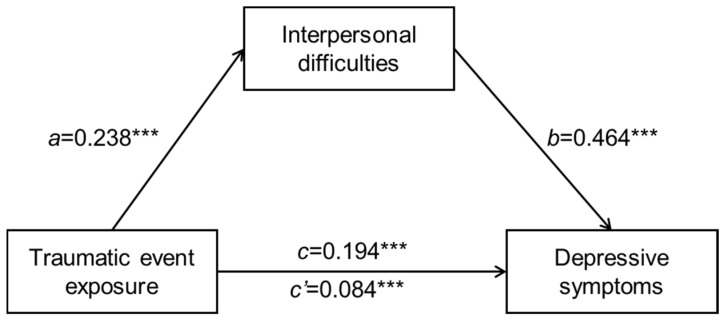
The mediating role of interpersonal difficulties in the association between traumatic event exposure and depressive symptoms. *** *p* < 0.001.

The mediating effect of interpersonal difficulties in the association between traumatic event exposure and depressive symptoms was examined using the PROCESS macro, controlling for sex, baseline age and residence. The significance of the indirect effect was tested using 5000 bootstrap samples. The results are presented in Figure 2 and Table 3. Traumatic event exposure positively predicted depressive symptoms (β = 0.194, 95% CI [0.169, 0.221], *p* < 0.001). Traumatic event exposure was positively associated with interpersonal difficulties (β = 0.238, 95% CI [0.216, 0.259], *p* < 0.001), which in turn were positively associated with depressive symptoms (β = 0.464, 95% CI [0.444, 0.484], *p* < 0.001). After interpersonal difficulties were included in the model, the direct association between traumatic event exposure and depressive symptoms remained significant (β = 0.084, 95% CI [0.061, 0.108], *p* < 0.001). The indirect effect through interpersonal difficulties was significant (β = 0.110, 95% CI [0.097, 0.124]), accounting for 56.82% of the total effect. These findings indicate that interpersonal difficulties partially mediated the association between traumatic event exposure and depressive symptoms.

### 3.5. Moderated Mediation Model Analysis

The moderating effect of only-child status on the relationships among traumatic event exposure, interpersonal difficulties, and depression is presented in Table 4. The interaction between traumatic event exposure and only-child status significantly predicted depressive symptoms (β = 0.067, *p* < 0.01), indicating that only-child status moderated the direct association between traumatic event exposure and depressive symptoms. In addition, the interaction between interpersonal difficulties and only-child status was also significant (β = −0.052, *p* < 0.05), indicating that only-child status moderated the association between interpersonal difficulties and depressive symptoms. Conditional indirect effects were significant for both non-only children (β = 0.115, 95% CI [0.101, 0.131]) and only children (β = 0.103, 95% CI [0.088, 0.118]). The index of moderated mediation was statistically significant but small (index = −0.013, 95% CI [−0.025, −0.001]), suggesting that the indirect association through interpersonal difficulties differed slightly by only-child status.

**Figure 3:**
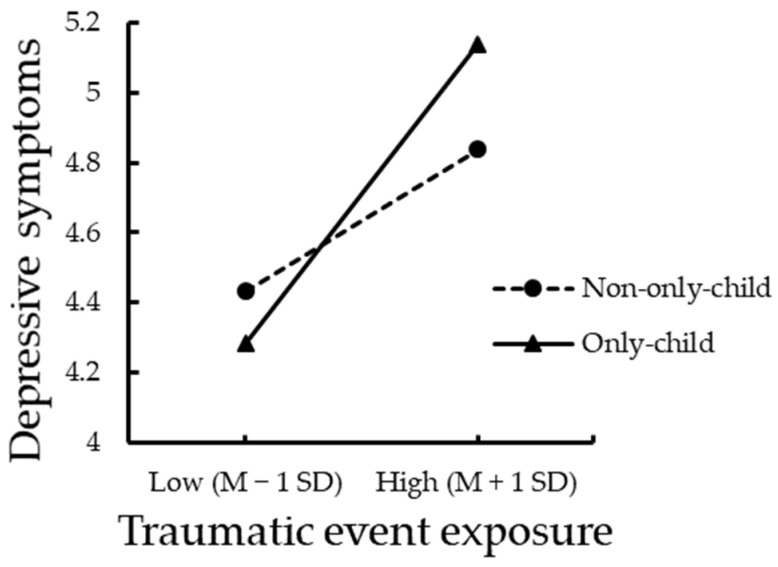
Moderating effect of only-child status on the association between traumatic event exposure and depressive symptoms.

**Figure 4:**
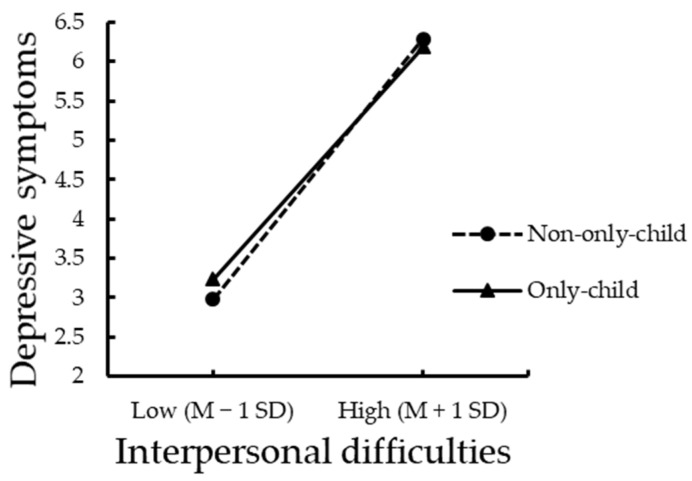
Moderating effect of only-child status on the association between interpersonal difficulties and depressive symptoms.

To further interpret the moderating effects, simple slope analyses were conducted. As shown in Figure 3, traumatic event exposure was positively associated with depressive symptoms in both groups. The association was stronger among only children (β = 0.127, *p* < 0.001) compared to non-only children (β = 0.060, *p* < 0.001). This suggests that students from single-child families may show greater depressive vulnerability when exposed to traumatic events. As shown in Figure 4, interpersonal difficulties were positively associated with depressive symptoms in both groups. The association was slightly stronger among students with siblings (β = 0.484, *p* < 0.001) than among students from single-child families (β = 0.432, *p* < 0.001).

## 4. Discussion

In this study, we used a large sample of Chinese university freshmen to examine the associations among traumatic event exposure, interpersonal difficulties, and depressive symptoms, as well as the moderating role of only-child status. The results showed that traumatic event exposure was positively associated with depressive symptoms. The indirect association through interpersonal difficulties was statistically significant, suggesting that students with greater traumatic event exposure tended to report more interpersonal difficulties, which were in turn associated with higher depressive symptoms. In addition, only-child status moderated the direct association between traumatic event exposure and depressive symptoms and the association between interpersonal difficulties and depressive symptoms. These findings highlight the importance of considering both interpersonal processes and family structure when understanding depressive symptoms among university students exposed to traumatic events.

### 4.1. Association Between Traumatic Event Exposure and Depressive Symptoms

The present study found that traumatic event exposure was positively associated with depressive symptoms among Chinese university freshmen, which is consistent with previous research linking trauma exposure to poor mental health outcomes (Infurna et al., 2016; Sezgin & Punamäki, 2021). In recent years, several studies have explored the relationship between traumatic events and depression, revealing how trauma experiences can affect an individual’s mental health. For instance, research has shown that traumatic events may make individuals more susceptible to depressive symptoms when exposed to stress (Southwick & Charney, 2012). The comorbidity between a history of trauma and depression has also received significant attention, with studies suggesting that trauma experiences may exacerbate the severity of depressive symptoms (Bedard-Gilligan et al., 2015). Furthermore, studies have shown a significant association between childhood trauma and adult depression. Individuals who experienced multiple traumas in childhood are at greater risk of developing chronic depressive symptoms in adulthood (Wiersma et al., 2009). Moreover, traumatic experiences can make individuals more vulnerable to depressive symptoms when encountering new trauma events, a phenomenon known as “stress sensitization” (Bandoli et al., 2017). The relationship between traumatic events and depressive symptoms is complex and multifaceted, highlighting the importance of further investigating the factors associated with depressive symptoms among college students. Future longitudinal and intervention studies are needed to determine whether addressing trauma-related factors may contribute to improving mental health outcomes.

### 4.2. Indirect Association Through Interpersonal Difficulties

This study provides evidence for a statistically significant indirect association between traumatic event exposure and depressive symptoms through interpersonal difficulties. Traumatic experiences may be related to how individuals perceive themselves and others, leading to mistrust, interpersonal sensitivity, avoidance, emotional withdrawal, and difficulties in forming or maintaining stable relationships (Chen et al., 2020; Goldstein et al., 2023). These interpersonal difficulties may be particularly important during the transition to university, when students are required to establish new peer networks and adapt to unfamiliar social environments. This finding is consistent with previous studies showing that trauma exposure is associated with interpersonal problems. Huh et al. reported that childhood trauma was related to adult interpersonal relationship problems among patients with depression and anxiety disorders (Huh et al., 2014). Goldstein et al. also found an association between trauma and interpersonal problems in a community mental health setting (Goldstein et al., 2023). In addition, Hernandez et al. demonstrated that childhood emotional abuse prospectively predicted interpersonal dependent stress, and that rejection sensitivity mediated this association, supporting the role of depressogenic interpersonal processes after early adversity (Hernandez et al., 2016). These findings provide theoretical and empirical support for the relationship between traumatic experiences and interpersonal difficulties.

Interpersonal difficulties may further contribute to depressive symptoms by influencing social support, loneliness, interpersonal stress, and emotional regulation. Poorer interpersonal relationship functioning may reduce access to emotional and instrumental support, increase feelings of isolation, and intensify negative self-evaluation. Previous research has shown that interpersonal trauma is associated with depression among adolescents and that interpersonal relationships may mediate this association (Chen et al., 2020). Longitudinal evidence also suggests that social relationships are related to later depression severity, indicating that interpersonal functioning is important for the course of depressive symptoms (Teo et al., 2013). Therefore, interpersonal communication, peer support, and social adaptation may represent important areas for future longitudinal and intervention research among university students with traumatic experiences.

### 4.3. Moderating Role of Only-Child Status

This study explored the moderating role of only-child status and found that association between traumatic event exposure and depressive symptoms was stronger among only children than among non-only children. Related research suggests that, due to the lack of support from siblings, only children may be more vulnerable and find it more difficult to regulate their emotions when facing stress and trauma (Liu & Jiang, 2021; Sun et al., 2025). In addition, only children in family environments often bear greater expectations and responsibilities, which may lead to a higher psychological burden when experiencing traumatic events. Studies show that only children may lack effective coping mechanisms when confronted with traumatic events, making them more vulnerable to depressive and anxiety symptoms (Cheng et al., 2020). Furthermore, given that childhood trauma has been found to partially mediate the development of depressive symptoms through dissociative symptoms in other studies (Fung & Cheung, 2024), and that only children may be more prone to dissociative responses due to limited peer interaction experiences, these factors may potentially contribute to differences in vulnerability observed among only children.

This study also found that the association between interpersonal difficulties and depressive symptoms was slightly stronger among non-only children than among only children. One possible explanation is that individuals with siblings may be more sensitive to the quality of interpersonal interactions because they have experienced more complex social exchanges within the family environment throughout development (Gozu & Newman, 2020; Yin et al., 2019; Zhou et al., 2023). However, it is important to note that the magnitude of this interaction effect was relatively small. Therefore, although the moderation effect reached statistical significance, its practical significance may be limited and should be interpreted with caution. Future studies should further examine whether factors such as sibling relationship quality, family cohesion, parent–child relationships, perceived social support, resilience-related resources, optimism, and cognitive resources account for these differences (Fabio et al., 2026; Suriano & Valentino, 2026).

### 4.4. Limitations and Future Prospects

Despite the valuable insights provided by this study, several limitations should be acknowledged. First, the cross-sectional design of the study prevents establishing causal relationships between traumatic event exposure, interpersonal difficulties, and depressive symptoms. Longitudinal studies are needed to better understand the temporal relationships among these variables. Second, although sex, age, and residence were included as covariates, several potentially important variables were not measured in this study. Personality traits, previous mental health history, socioeconomic status, parent–child relationship quality, family cohesion, coping strategies, perceived social support, trauma severity, and specific characteristics of traumatic events may partly explain or modify the observed associations. Future studies should include a broader range of individual, family, and social-contextual variables to clarify whether the observed associations remain robust after accounting for these factors. Additionally, the reliance on self-reported data may introduce biases such as social desirability and recall biases, which could affect response accuracy. Future research should incorporate multiple data sources, such as clinical interviews or behavioral assessments, to improve reliability. Another limitation is the focus on Chinese university students, which may limit the generalizability of the findings to other cultural or demographic groups. Future studies should explore similar relationships in diverse populations to evaluate the cross-cultural applicability of the findings. Furthermore, although the STEQ scale was developed through reference to previous trauma exposure instruments, expert review, and pilot testing, it remains a newly developed measure. The present study did not evaluate its factor structure or convergent validity with established trauma exposure instruments. Future studies are needed to further validate the psychometric properties of the STEQ across different populations.

## 5. Conclusions

This study suggests that traumatic event exposure is associated with depressive symptoms among Chinese university freshmen both directly and indirectly through interpersonal difficulties. Only-child status further moderates these associations: the association between traumatic event exposure and depressive symptoms was stronger among only-children, whereas the association between interpersonal difficulties and depressive symptoms was slightly stronger among students with siblings. These findings suggest that traumatic event exposure, interpersonal difficulties, and family structure are associated with depressive symptoms among Chinese university freshmen. Future longitudinal and intervention studies are needed to clarify temporal relationships and evaluate potential intervention targets.

## Figures and Tables

**Table 1:** Demographic characteristics of 8079 first-year university students.

Variables	*n*	Traumatic Event Exposure		Depressive Symptoms		Interpersonal Difficulties	
		Mean ± SD	*t*/*p*	Mean ± SD	*t*/*p*	Mean ± SD	*t*/*p*
Gender							
Male	3225	1.29 ± 1.30	*t* = 2.017	4.52 ± 3.61	*t* = −2.947	5.49 ± 5.46	*t* = −3.162
Female	4854	1.23 ± 1.26	(*p* = 0.044)	4.75 ± 3.27	(*p* = 0.003)	5.88 ± 5.11	(*p* = 0.002)
Residence							
Urban	2995	1.22 ± 1.28	*t* = −2.078	4.61 ± 3.49	*t* = −0.939	5.43 ± 5.17	*t* = −3.875
Rural	5084	1.28 ± 1.28	(*p* = 0.038)	4.69 ± 3.36	(*p* = 0.348)	5.90 ± 5.30	(*p* < 0.001)
Only-child							
Yes	3087	1.22 ± 1.22	*t* = −2.013	4.62 ± 3.49	*t* = −0.785	5.48 ± 5.27	*t* = −3.227
No	4992	1.28 ± 1.32	(*p* = 0.044)	4.68 ± 3.37	(*p* = 0.432)	5.87 ± 5.24	(*p* = 0.001)

**Table 2:** Means, standard deviations, and bivariate correlations among study variables.

	M	SD	Traumatic Event Exposure	Interpersonal Difficulties	Depressive Symptoms
Traumatic event exposure	1.25	1.28	1		
Interpersonal difficulties	5.72	5.26	0.237 **	1	
Depressive symptoms	4.66	3.41	0.193 **	0.481 **	1

Note: ** *p* < 0.01.

**Table 3:** Breakdown of total effect, direct effect and mediated effect.

Items	Effect	95% CI	Relative Effect
Mediated effect	0.110 ***	[0.097, 0.124]	56.82%
Direct effect	0.084 ***	[0.061, 0.108]	43.18%
Total effect	0.194 ***	[0.169, 0.221]	

Note: Effects are reported as standardized coefficients, *** *p* < 0.001.

**Table 4:** Moderated mediation analysis.

Variable	Model 1 Interpersonal Difficulties			Model 2 Depressive Symptoms		
	β	*t*	95% CI	β	*t*	95% CI
Traumatic event exposure	0.238	21.549 ***	[0.216, 0.259]	0.060	4.761 ***	[0.035, 0.085]
Interpersonal difficulties				0.484	37.268 ***	[0.458, 0.509]
Only-child status				0.021	1.036	[−0.019, 0.061]
Traumatic event exposure * Only-child status				0.067	3.130 **	[0.025, 0.109]
Interpersonal difficulties * Only-child status				−0.052	−2.485 *	[−0.093, −0.011]
*R* ^2^	0.056			0.242		
*F*	464.354 ***			494.869 ***		

Note: * *p* < 0.05; ** *p* < 0.01; *** *p* < 0.001.

## Data Availability

The original contributions presented in this study are included in the article/Supplementary Materials. Further inquiries can be directed to the corresponding authors.

## Supplementary Materials

The following supporting information can be downloaded at: https://www.mdpi.com/article/10.3390/bs16071243/s1, Table S1: Frequencies of potentially traumatic or severe adverse events assessed by the STEQ.

## Abbreviations

The following abbreviations are used in this manuscript:

PHQ-9	Patient Health Questionnaire-9
CDSIR	Comprehensive Diagnostic Scale of Interpersonal Relationships
STEQ	Severe Traumatic Events Questionnaire
VIF	Variance Inflation Factor

## Appendix A. Items of the Severe Traumatic Events Questionnaire

1. In your life, have the following events occurred: The death of a grandparent
2. In your life, have the following events occurred: Death of father or mother
3. In your life, have the following events occurred: Death of sibling
4. In your life, have the following events occurred: Have had a serious illness
5. In your life, have the following events occurred: Natural disasters such as fires, floods, and earthquakes
6. In your life, have the following events occurred: Suffered a physical attack or assault
7. In your life, have the following events occurred: Have witnessed someone seriously disabled or killed
8. In your life, have the following events occurred: When I was a child, I was physically abused by my parents
9. In your life, have the following events occurred: When I was a child, I suffered emotional/mental abuse from the parent
10. In your life, have the following events occurred: When I was a child, I was neglected and ignored by my parents
11. In your life, have the following events occurred: Gender discrimination in the family
12. In your life, have the following events occurred: Gender discrimination in schools
13. In your life, have the following events occurred: Campus bullying (victim)
14. In your life, have the following events occurred: Campus bullying (violators)
15. In your life, have the following events occurred: Be invited or asked to do sex-related activities
16. In your life, have the following events occurred: Involuntary kissing or hugging with sexual intent
17. In your life, have the following events occurred: Involuntary nudity or naked sex organs
18. In your life, have the following events occurred: Involuntary touching or being touched on private parts
19. In your life, have the following events occurred: Involuntary sexual intercourse
20. In your life, have the following events occurred: Voluntary intercourse
21. In your life, have the following events occurred: Being raped
22. In your life, have the following events occurred: Adopted/fostered
23. In your life, have the following events occurred: Left-behind children (parents are away from home)
